# Supplementary material for: Predicting Long-Term Childhood Survival of Newborns with Congenital Heart Defects: A Population-Based, Prospective Cohort Study (EPICARD)
Source: J Clin Med. 2024 Mar 12;13(6):1623. doi: 10.3390/jcm13061623 (PMC10970958; doi:10.3390/jcm13061623)
Supplement: Supplementary file 1 [file jcm-13-01623-s001.zip › jcm-2845054-supplementary.pdf]

Figure S1. Predicted instantaneous hazard in different ACC-CHD groups estimated by model 2 with ACC-CHD and other predictors at their average

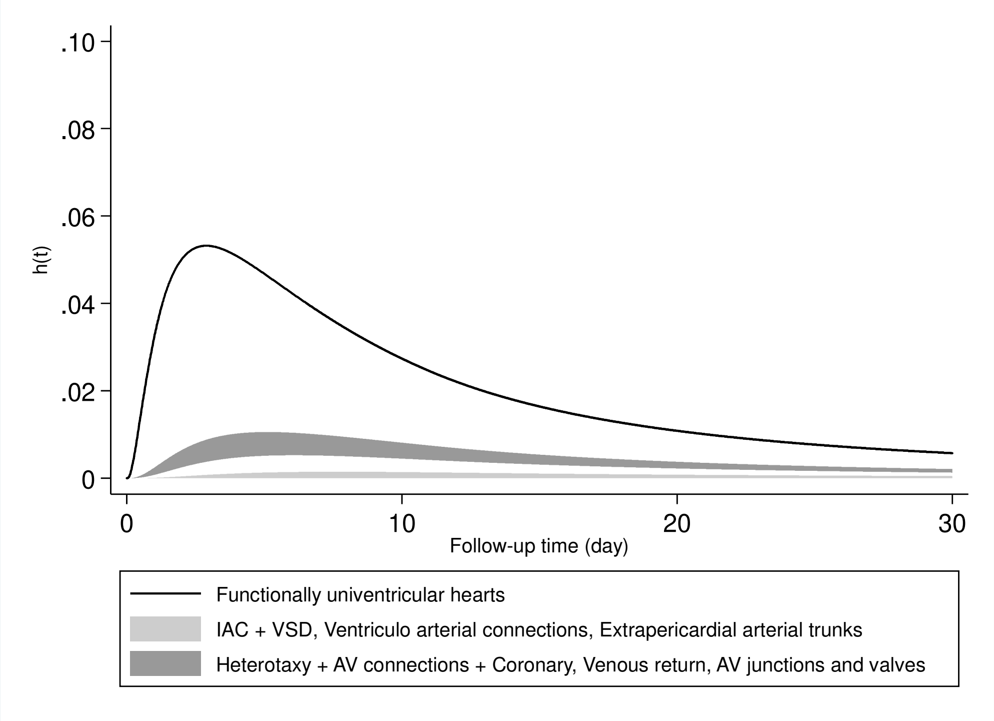

Figure S2. Kaplan-Meier plots for 8 years of follow-up in different categories of ACC-CHD

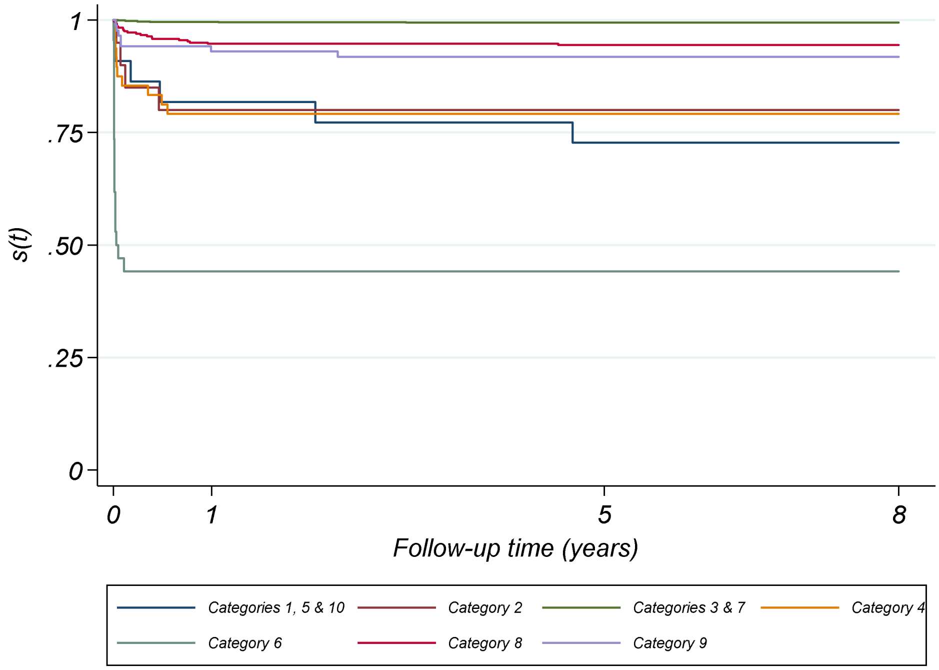

Figure S3. Goodness of fit – Model 2

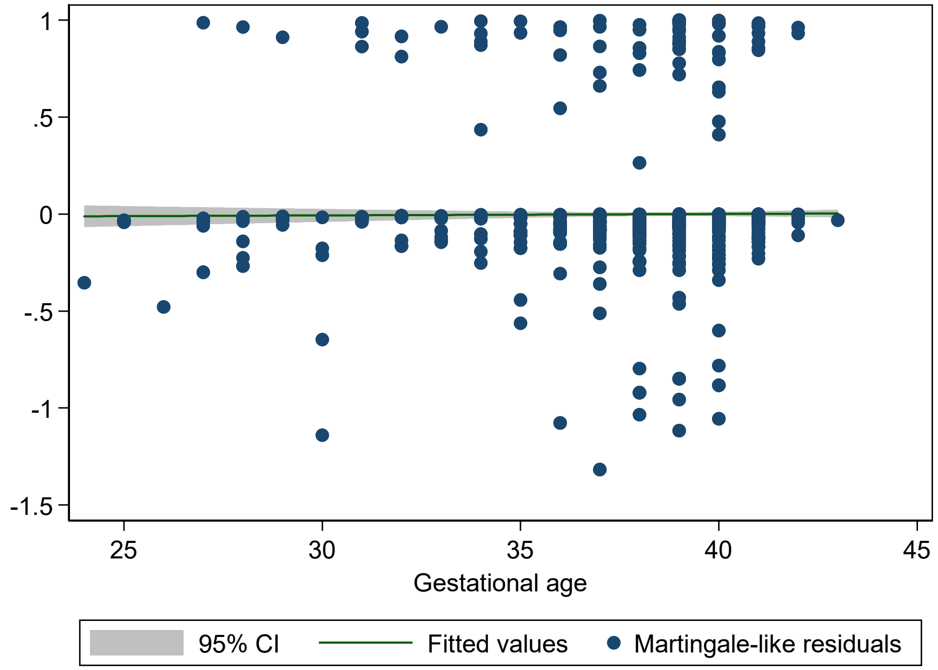

Figure S4. Estimates of survivor function in different ACC-CHD categories – Model 1

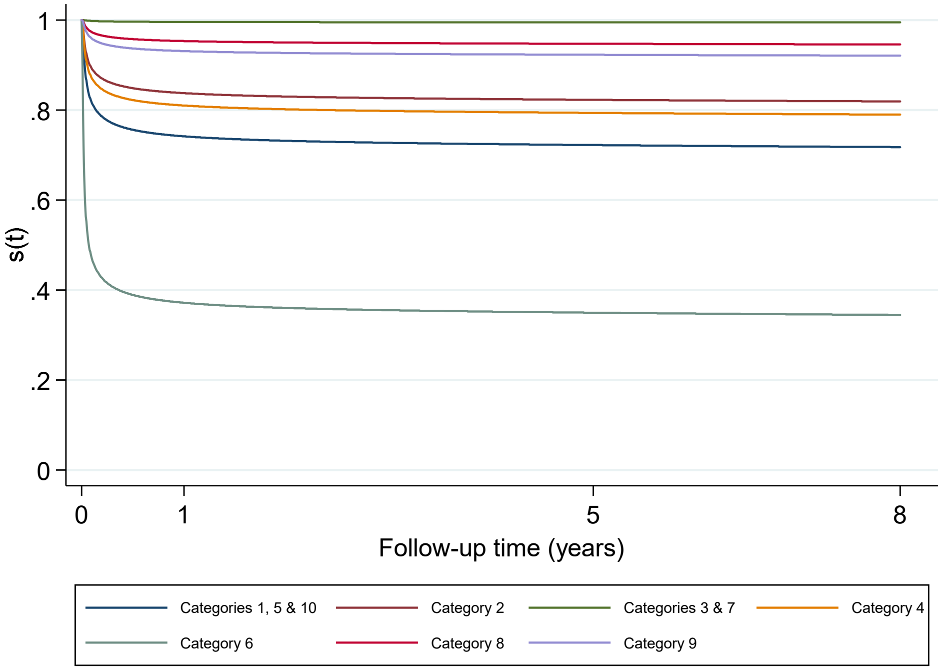

Figure S5. 8-year baseline hazard function in different ACC-CHD categories – Model 1

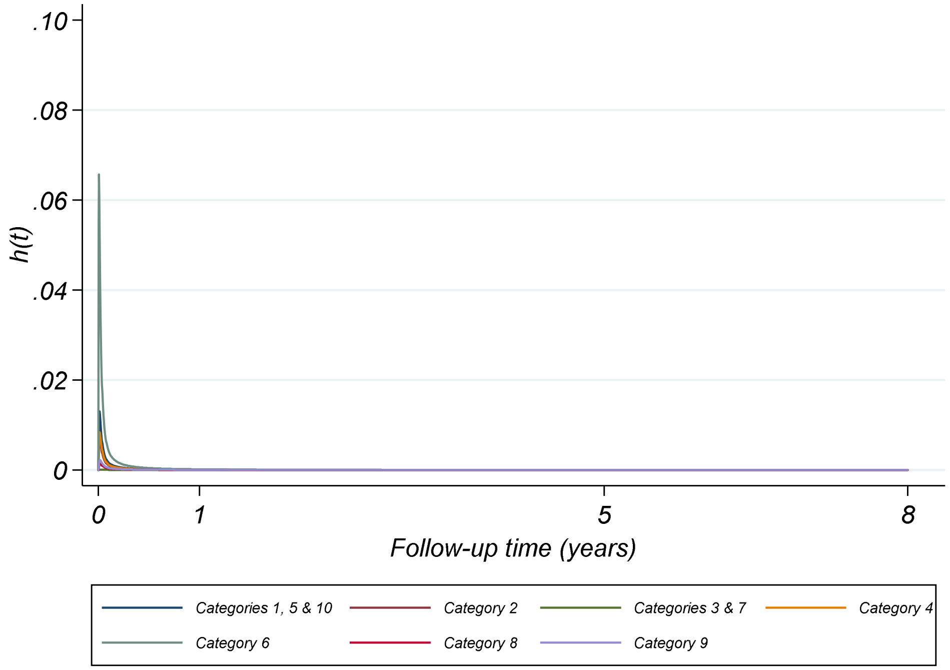

Figure S6. Estimates of baseline hazard function for ACC-CHD categories – Model 2

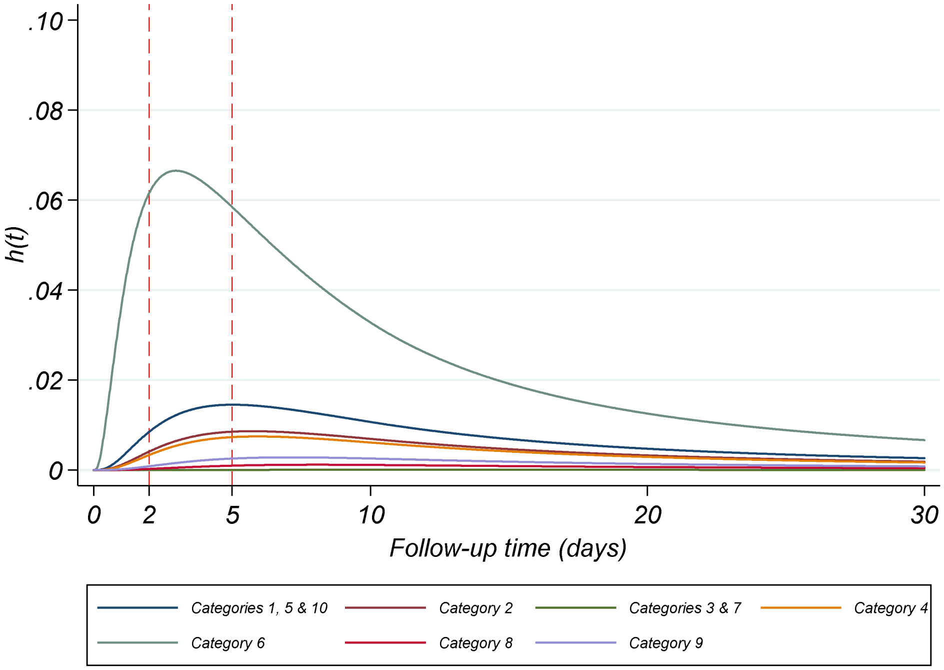

Figure S7. 8-year baseline hazard function in different ACC-CHD categories – Model 2

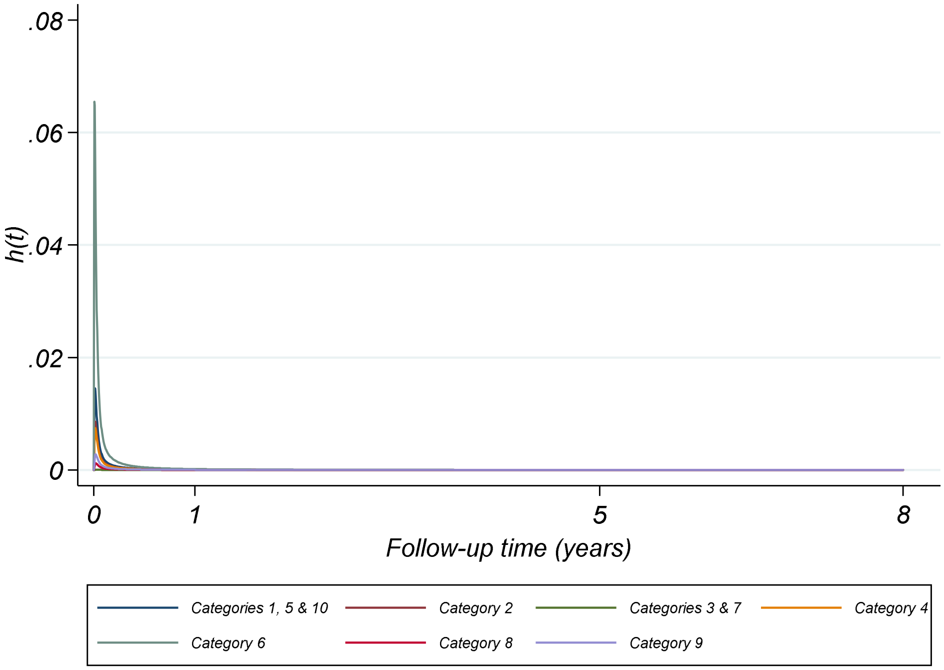

Figure S8. Survival probability in men and women general population

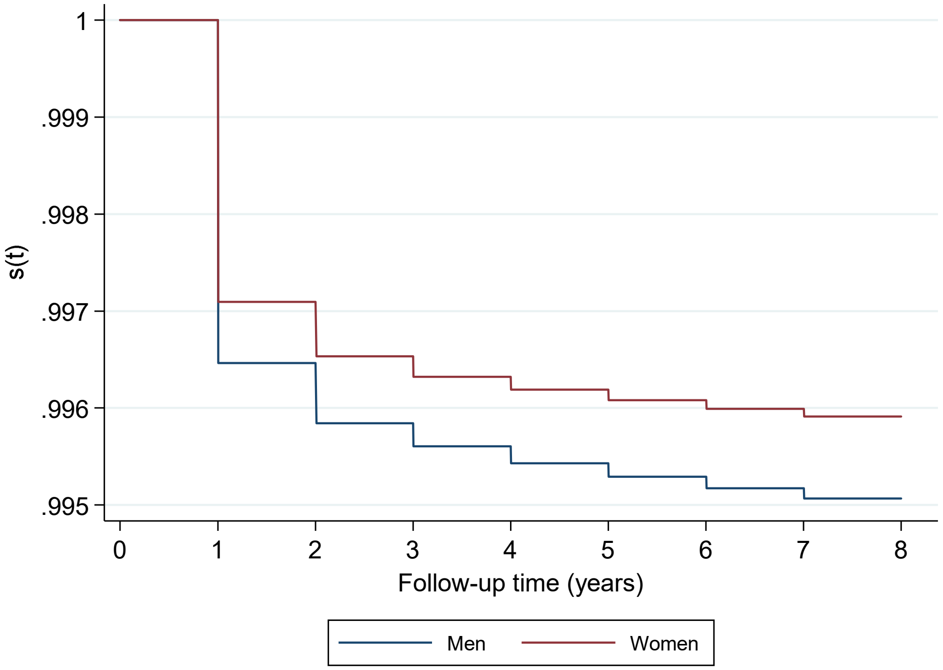

Figure S9. Comparison of survivor estimate of ACC-CHD categories with general population

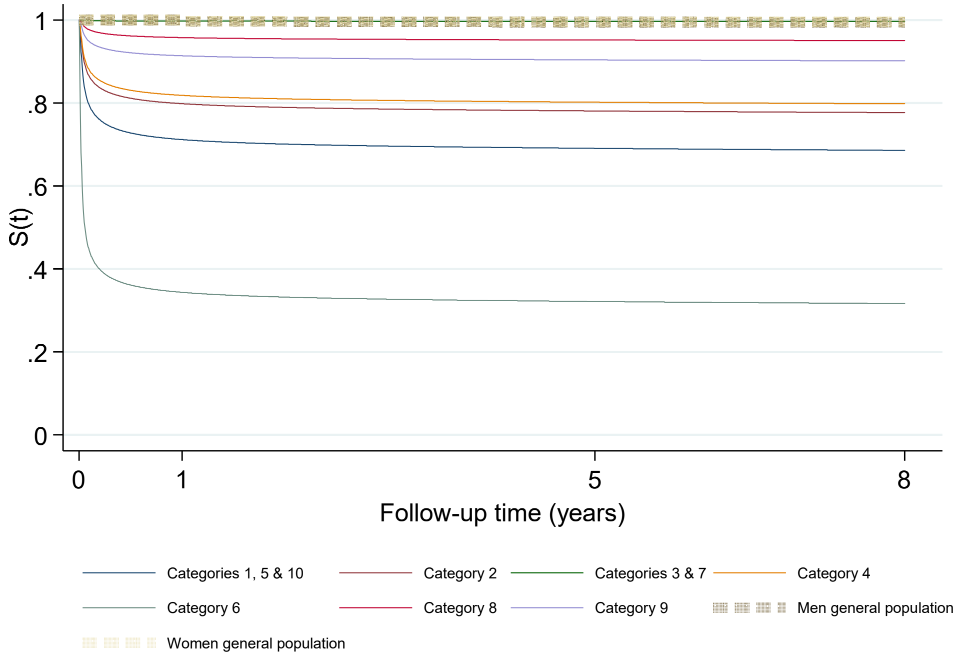

Figure S10. Comparison of survivor estimate of ACC-CHD merged categories 3 & 7 with men general population

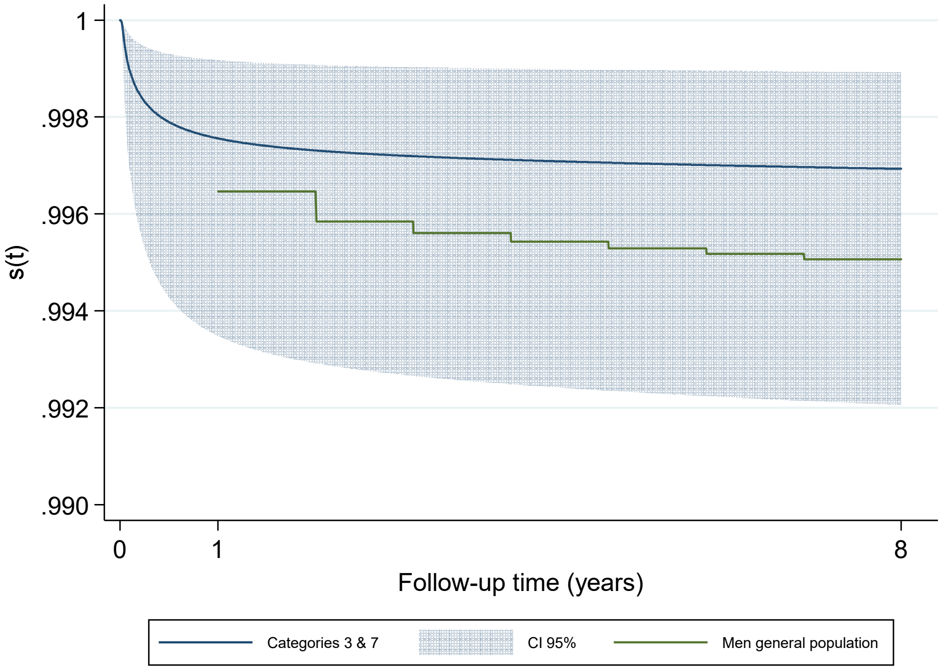

Figure S11. Comparison of survivor estimate of ACC-CHD merged categories 3 & 7 with women general population

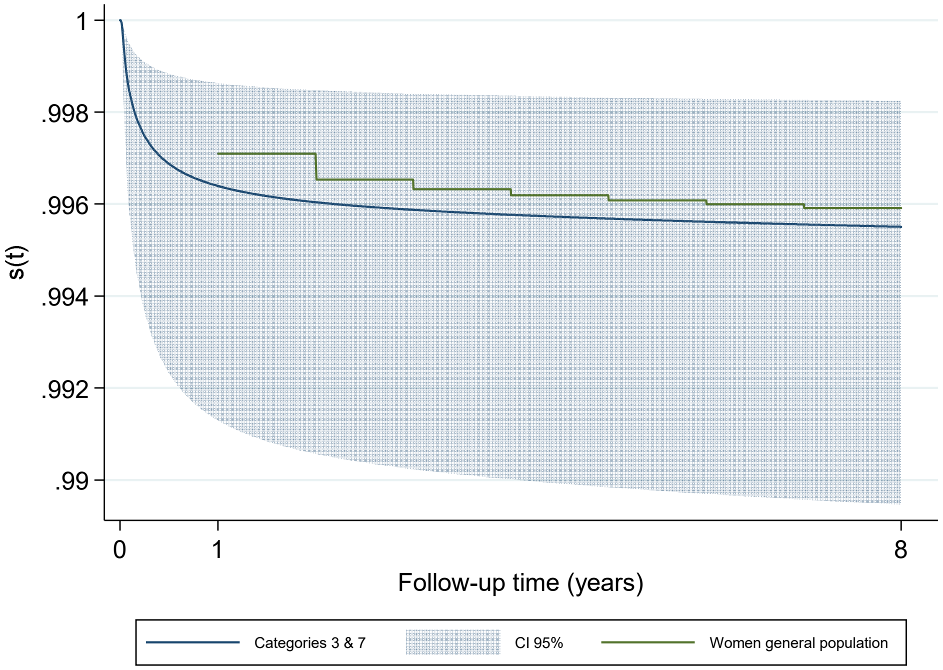

Table S1. Descriptive characteristics of study population in different ACC-CHD groups, N (% of column) or mean ± SD

	Gro up 1	Gro up 2	Gro up 3	Gro up 4	Gro up 5	Gro up 6	Gro up 7
Female	8 (36.4)	12 (60)	745 (56.8)	23 (47.9)	12 (35.3)	183 (50.8)	35 (40.7)
Gestation al age (week)	38.2 ± 3.4	39.3 ± 1.5	38.5 ± 2.4	38.5 ± 2.8	38.5 ± 2.2	38.1 ± 2.7	38.8 ± 1.8
Prematuri ty†	3 (13.6)	1 (5.0)	152 (11.6)	8 (16.7)	4 (11.8)	63 (17.6)	9 (10.5)
SGA‡	2 (9.1)	2 (10.0)	133 (10.2)	5 (10.4)	7 (20.6)	45 (12.6)	11 (12.8)
Surgery§	18 (81.8)	15 (75)	84 (6.4)	19 (39.6)	16 (47.1)	153 (42.5)	77 (89.5)
8-year survival	15 (68.2)	16 (80.0)	1303 (99.4)	37 (77.1)	15 (44.1)	340 (94.4)	79 (91.9)

*Group 1: Heterotaxy, Atrioventricular (AV) connections and Coronary arteries anomalies, Group 2: Venous return anomalies, Group 3: Interatrial communications and Ventricular septal defects, Group 4: AV junctions and valves, Group 5: Functionally univentricular hearts, Group 6: Ventriculo-arterial connections, Group 7: Extrapericardial arterial trunks
† < 37 weeks of gestation
‡ < 10th percentile of Audipog's curve
§ surgery during 1st year of life

Table S2. Anatomic and Clinical Classification of CHD (ACC-CHD)

1 - Heterotaxy, including isomerism and mirror-imagery

2 - Anomalies of the venous return

3 - Anomalies of the atria and interatrial communications

4 - Anomalies of the atrioventricular junctions and valves

5 - Complex anomalies of atrioventricular connections

6 - Functionally univentricular hearts

7 - Ventricular septal defects

8 - Anomalies of the ventricular outflow tracts (ventriculo-arterial connections)

9 - Anomalies of the extrapericardial arterial trunks

10 - Congenital anomalies of the coronary arteries

Table S3. 8-year mortality rate in different categories of ACC-CHD variable

ACC-CHD*	N (%)	Deaths (%)**	Merged	N (%)	Deaths (%)
**1**	6 (0.32)	2 (33.33)	**1, 5 & 10**	22 (1.17)	6 (27.27)
**2**	20 (1.06)	4 (20.00)	**2**	20 (1.06)	4 (20.00)
**3**	126 (6.70)	1 (0.79)	**3 & 7**	1311 (69.70)	7 (0.53)
**4**	48 (2.55)	10 (20.83)	**4**	48 (2.55)	10 (20.83)
**5**	7 (0.37)	3 (42.86)			
**6**	34 (1.81)	19 (55.88)	**6**	34 (1.81)	19 (55.88)
**7**	1185 (63.00)	6 (0.51)			
**8**	360 (19.14)	20 (5.56)	**8**	360 (19.14)	20 (5.56)
**9**	86 (4.57)	7 (8.14)	**9**	86 (4.57)	7 (8.14)
**10**	9 (0.48)	1 (11.11)			

*ACC-CHD categories: 1,5 & 10 (Other CHD); 2 (Venous return); 3 & 7 (IAC & VSD); 4 (Atrioventricular junction & valve); 6 (FUH); 8 (Ventricular outflow tract); 9 (Extrapericardial arterial trunks)
**Percentage of all deaths in each group

Table S4. Non-parametric survival and hazard estimates for 8 years of follow-up

**ACC-CHD***	**Kaplan-Meier survival estimate****	**Nelson-Aalen cumulative hazard**
**1, 5 & 10**	0.73 [0.49; 0.87]	0.31 [0.14; 0.69]
**2**	0.80 [0.55; 0.92]	0.22 [0.08; 0.58]
**3 & 7**	0.995 [0.989; 0.997]	0.005 [0.003; 0.011]
**4**	0.79 [0.65; 0.88]	0.23 [0.12; 0.43]
**6**	0.44 [0.27; 0.60]	0.76 [0.47; 1.21]
**8**	0.94 [0.91; 0.96]	0.06 [0.04; 0.09]
**9**	0.92 [0.84; 0.96]	0.08 [0.04; 0.18]

* ACC-CHD categories: 1,5 & 10 (Other CHD); 2 (Venous return); 3 & 7 (IAC & VSD); 4 (Atrioventricular junction & valve); 6 (FUH); 8 (Ventricular outflow tract); 9 (Extrapericardial arterial trunks)
**p-value of Wilcoxon test for equality of survivor function: <0.001

Table S5. Flexible parametric survival models

	Model 1 *			Model 2 **		
	**Coefficient**	**P >|z|**	**95% CI**	**Coefficient**	**P >|z|**	**95% CI**
**ACC-CHD*** **						
1, 5 & 10	1.98	<0.001	[1.40 - 2.58]	2.26	<0.001	[1.62 - 2.89]
2	1.65	<0.001	[0.98 - 2.31]	1.98	<0.001	[1.27 - 2.68]
4	1.76	<0.001	[1.29 - 2.22]	1.90	<0.001	[1.42 - 2.39]
6	2.96	<0.001	[2.49 - 3.43]	3.22	<0.001	[2.71 - 3.73]
8	0.96	<0.001	[0.63 - 1.29]	1.09	<0.001	[0.73 - 1.45]
9	1.15	<0.001	[0.69 - 1.61]	1.45	<0.001	[0.92 - 1.98]
**Gender**						
Female				0.13	0.332	[-0.13 - 0.39]
**SGA**						
Yes				0.28	0.109	[-0.06 - 0.62]
**Surgery**						
Yes				-0.27	0.078	[-0.58 - 0.03]
**Gestational Age**				-0.08	<0.001	[-0.12 - -0.04]
**_rcs1†**	0.17	<0.001	[0.14, 0.21]	0.19	<0.001	[0.15 - 0.23]
**_rcs2†**	0.08	<0.001	[0.06 - 0.11]	0.09	<0.001	[0.07 - 0.12]
**_rcs3†**	-0.01	0.046	[-0.02 - 0.00]	-0.01	0.025	[-0.03 - -0.001]
**Constant**	-2.59	<0.001	[-2.85 - -2.33]	0.20	0.798	[-1.30 - 1.69]

* Model 1: with only ACC-CHD variable, AIC: 715.02, BIC: 770.42; ** Model 2: with all predictor variables, AIC: 700.44, BIC: 777.92

*** ACC-CHD categories: 1,5 & 10 (Other CHD); 2 (Venous return); 3 & 7 (IAC & VSD); 4 (Atrioventricular junction & valve); 6 (FUH); 8 (Ventricular outflow tract); 9 (Extrapericardial arterial trunks)
